# Early treatment with tecovirimat against mpox disease in cynomolgus macaques

**DOI:** 10.1016/j.ebiom.2026.106526

**Published:** 2026-10-21

**Authors:** Annika Rössler, Malika Aid, Jose Ayala-Bernot, Jinyan Liu, Andrew T. Russo, Douglas W. Grosenbach, Candace Lovejoy, Anthony Cook, Hanne Andersen, Dennis E. Hruby, Dan H. Barouch

**Affiliations:** a Center for Virology and Vaccine Research, Beth Israel Deaconess Medical Center, Harvard Medical School, Boston, MA 02215, USA; b SIGA Technologies, Inc., Corvallis, OR 97333, USA; c Bioqual, Rockville, MD 20852, USA

**Keywords:** Mpox, Tecovirimat, TPOXX, Antiviral, Poxviruses

## Abstract

**Background:**

Tecovirimat was developed as an antiviral countermeasure against orthopoxviruses and approved by the US Food and Drug Administration Animal Rule based on efficacy in preclinical studies and clinical pharmacokinetic and safety trials. However, recent randomised clinical trials in patients with mpox who initiated tecovirimat an average of 6–9 days after onset of symptoms did not show improvement in time to resolution of skin lesions. Here, we evaluated whether timing of tecovirimat initiation influenced antiviral efficacy following mpox infection in nonhuman primates and defined the therapeutic window for treatment initiation.

**Methods:**

In this randomised preclinical study, 32 cynomolgus macaques were challenged intravenously with 1 × 10^6^ TCID_50_ mpox clade IIb virus. Oral tecovirimat (10 mg/kg, once daily for 14 days) treatment was initiated on Day 2 (post-exposure and pre-symptomatic), Day 5 (early symptomatic), or Day 9 (peak symptomatic) after mpox challenge, or no treatment was given as a control group. The primary study endpoint was area under the curve (AUC) and peak numbers of progressive skin lesions. The secondary endpoint was AUC and peak levels of plasma viral loads. Additional analyses included total skin lesions, adaptive immune responses, and transcriptomic signatures.

**Findings:**

Treatment adherence was 100%. All tecovirimat-treated animals survived to study completion on Day 28, whereas two untreated control animals required early euthanisation on Days 16 and 17. Tecovirimat efficacy was time-dependent. Progressive skin lesion AUC was significantly lower when treatment was initiated on Day 2 (*P* < 0.0001) or Day 5 (*P* = 0.0359) compared with untreated controls, but treatment was largely ineffective when initiated on Day 9 (*P* > 0.99).

**Interpretation:**

In this randomised preclinical study, tecovirimat demonstrated antiviral therapeutic efficacy but only when initiated early prior to peak viraemia. These findings emphasise the importance of early initiation of treatment and define the therapeutic window for optimal tecovirimat treatment in nonhuman primates.

**Funding:**

This study was supported by SIGA Technologies.

**Research in context**

**Evidence before this study**

We searched PubMed for preclinical challenge models and clinical trials published between Jan 1, 2018, and July 27, 2026, using search terms including “tecovirimat”, “TPOXX”, “mpox”, and “clinical trial”. Preclinical studies demonstrated robust efficacy when tecovirimat was administered early post–challenge, supporting US FDA Animal Rule approval. However, two phase 3 randomised controlled trials (PALM007 and STOMP) failed to meet their primary endpoint of time to lesion resolution. This discordance may reflect differences in treatment timing, as clinical trial participants were enrolled days after symptom onset, whereas in animal studies treatment was initiated early post-exposure. We hypothesised that tecovirimat efficacy against mpox is dependent on disease stage and timing of treatment initiation.

**Added value of this study**

This randomised preclinical study defines the treatment initiation window required for tecovirimat efficacy against mpox in nonhuman primates. Our data demonstrate that tecovirimat treatment must be initiated rapidly after mpox infection and before peak viraemia and peak skin lesions to achieve optimal therapeutic efficacy. Moreover, our findings highlight the importance of selecting endpoint criteria that involve reduction of disease progression rather than improved skin healing.

**Implications of all the available evidence**

This study provides a rationale for the discordance between successful animal studies and the negative results of the STOMP and PALM007 clinical trials. The findings suggest that timing of treatment initiation and endpoint selection are central to evaluating tecovirimat efficacy in mpox and highlight that future clinical trials should consider tecovirimat for post-exposure prophylaxis or early therapeutic intervention, with carefully selected endpoints that assess prevention of disease progression and viral dissemination.

## Introduction

Tecovirimat (ST-246, TPOXX) was developed as an antiviral countermeasure against orthopoxviruses, initially in the setting of smallpox preparedness and biodefence.^1^ Because variola virus has been eradicated and human efficacy trials are not feasible, tecovirimat was advanced under the US Food and Drug Administration Animal Rule, which permits approval on the basis of efficacy in well-characterised animal models together with safety and pharmacokinetic data in humans.^2^, ^3^, ^4^

Tecovirimat is administered orally, typically once daily for 14 days, and targets the conserved poxvirus F13/VP37 protein required for formation of extracellular enveloped virions and viral dissemination.^5^, ^6^, ^7^, ^8^, ^9^, ^10^ Preclinical studies have demonstrated broad antiviral activity of tecovirimat against multiple orthopoxviruses, including rabbitpox, cowpox, vaccinia, monkeypox, and variola, with substantial therapeutic efficacy observed across small and large animal challenge models, including nonhuman primates, particularly when treatment was initiated early.^9^, ^11^, ^12^, ^13^, ^14^, ^15^ Antiviral treatment did not appear to diminish the development of protective immunity, as nonhuman primates treated with tecovirimat in the setting of ACAM2000 vaccination remained protected against lethal mpox challenge.^16^

Clinical Phase 1 (dose-escalation) and Phase 2 (repeated-dosage) and extended pharmacokinetic and safety studies in healthy individuals have shown that oral tecovirimat given twice daily for 14 days was generally well tolerated and achieved systemic drug levels at or above levels associated with antiviral activity in animal models.^7^, ^8^, ^17^ These findings established the pharmacokinetic framework for regulatory approval of tecovirimat for smallpox and also provided the rationale to investigate its activity against other orthopoxviruses such as mpox.^3^, ^8^

The global clade IIb mpox outbreak beginning in 2022 provided the opportunity to conduct clinical trials to evaluate whether tecovirimat would provide therapeutic efficacy against mpox in humans. However, the two randomised clinical trials, STOMP (A5418) and PALM007, did not achieve their primary clinical endpoints. In STOMP, tecovirimat did not improve the primary endpoint of time to resolution of skin lesions in patients with clade II mpox and also did not significantly improve pain, complete lesion healing, or viral DNA clearance compared with placebo.^10^ In PALM007, tecovirimat did not reduce the time to resolution of skin lesions in patients with clade I mpox.^18^

Much of the preclinical evidence supporting tecovirimat were studies involving early treatment against lethal challenges.^9^, ^11^, ^12^, ^13^, ^14^, ^15^ By contrast, the clinical trials enrolled participants with clinically established disease and used lesion-resolution endpoints, although STOMP permitted treatment initiation based on clinical suspicion prior laboratory confirmation. In this study, we sought to determine whether tecovirimat efficacy was dependent on timing of initiation of therapy and to define the therapeutic window for initiation of therapy. We evaluated initiation of tecovirimat treatment at pre-symptomatic, early symptomatic, and peak symptomatic stages in a nonlethal intravenous clade IIb mpox challenge model in cynomolgus macaques, which reflects the typical self-limited course of clade IIb mpox disease in most humans.^19^, ^20^

## Methods

### Ethics

Animals were housed at Bioqual, Inc. (Rockville, MD), and all procedures were conducted under animal biosafety level 3 containment in accordance with approved Animal Care and Use Committee protocol (BIOQUAL protocol no. 25-051) and following recommendations from the Guide for the Care and Use of Laboratory Animals of the Office of Animal Welfare, National Institutes of Health (NIH) and the Animal Welfare Act of the US Department of Agriculture, in Association for the Assessment and Accreditation of Laboratory Animal Care International-accredited facilities. The study is reported in accordance with the ARRIVE guidelines.

### Study design and animals

Thirty-two female and male cynomolgus macaques (*Macaca fascicularis*) were randomly allocated with balancing for sex and body weight into three tecovirimat intervention groups (n = 8/group) or one untreated control group (n = 8). The group size was determined prospectively by power analysis using lesion count and plasma viral load data from prior nonhuman primate studies,^21^ providing approximately 80% power at a two-sided α of 0.05. Each group included 4–5 female and 3–4 male animals, and the mean body weight per group ranged from 6.47 to 6.81 kg. A schematic overview of the study design, including the treatment and clinical evaluation schedule is shown in Fig. 1. Baseline blood samples were collected 6 weeks prior to mpox virus challenge Detailed descriptions of all study methods are provided in the Supplementary material.

**Fig. 1:**
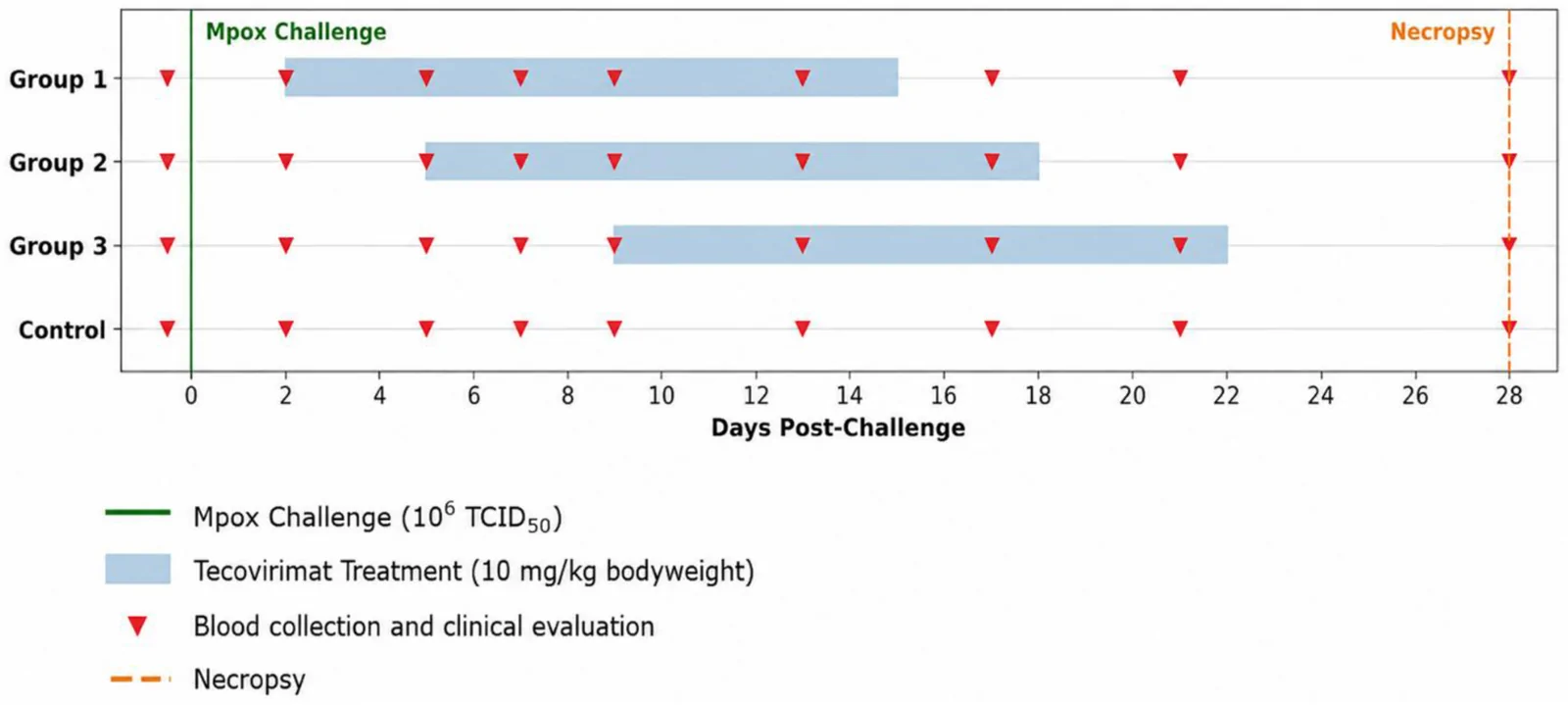
**Study design.** Thirty-two cynomolgus macaques were randomly allocated into three intervention groups andacontrol group (n = 8 per group) and were infected intravenously on Day 0 with 1 × 10^6^ TCID50 mpox clade IIb (strain hMPXV/USA/MA001/2022) virus. Tecovirimat was administered orally at 10 mg/kg bodyweight once daily for 14 consecutive days, beginning on Day 2 (Group 1; pre-symptomatic), Day 5 (Group 2; early symptomatic), or Day 9 (Group 3; peak symptomatic). Control animals received no treatment. Blood samples were collected longitudinally for plasma viral load quantification and transcriptomic analyses. Clinical assessments, including counting of skin lesions, were performed at defined intervals throughout the study. Animals were euthanised on Day 28 or if they met predefined humane endpoint criteria in accordance with IACUC protocols.

### Mpox challenge virus

All animals were infected intravenously with 1 × 10^6^ TCID_50_ mpox clade IIb virus (hMPXV/USA/MA001/2022; lineage B.1, Clade IIb), originally sourced from BEI Resources (NR-58622). The virus was propagated on MA-104 cells (Clone 1; ATCC CRL 2378.1; RRID: CVCL_3846) to generate working stocks at Bioqual, Inc., and titre was determined by plaque assay on Vero E6 cells (ATCC CRL-1586/Vero C1008; RRID: CVCL_0574). The challenge inoculum was prepared in PBS at 1 × 10^6^ TCID50 in a total volume of 1 mL intravenous infusion per animal. MA-104 Clone 1 cells (ATCC CRL-2378.1; RRID:CVCL_3846) are reported to be misidentified with respect to species origin (African green monkey rather than rhesus macaque); this is not expected to affect their use in this study as a permissive substrate for virus propagation. The cell lines used in this study were not independently validated and were not routinely tested for mycoplasma.

### Tecovirimat intervention

To define the therapeutic window for tecovirimat efficacy, treatment was initiated on Day 2 (Group 1; post-exposure pre-symptomatic disease prior to viraemia), Day 5 (Group 2; early symptomatic disease with rising viraemia), or Day 9 (Group 3; peak symptomatic disease with peak viraemia) following Mpox virus challenge. Treatment was administered once daily by oral gavage (10 mg/kg bodyweight) for 14 consecutive days aligning previous nonhuman primate pharmacokinetic bridging studies with the approved human oral dosing regimen.^8^ The control animals received no treatment.

### Study endpoints

Primary study endpoint was progressive skin lesion burden, evaluated by area under the curve (AUC) and peak numbers of progressive skin lesions. Secondary endpoints were plasma viral burden (AUC and peak levels). Both parameters were assessed at predefined timepoints over the course of the study (Fig. 1), while humoral and cellular immune responses were evaluated after disease resolution.

### Clinical assessments of primary study endpoint

Skin lesions were enumerated and staged by trained personnel who were not blinded to treatment group, using prespecified criteria (stage 1, macule; stage 2, papule; stage 3, vesicle; stage 4, pustule; stage 5, scab).^22^ Progressive lesions were defined as lesions that advanced beyond stage 1.

### Plasma viral loads analysis of secondary study endpoint

Total viral DNA was quantified by real-time polymerase chain reaction (qRT-PCR) targeting the F3L gene as previously described.^21^, ^23^ More detailed information is provided in the Supplementary material and Supplementary Table S1.

### Humane endpoint criteria

Animals were monitored daily for 28 days after challenge. Humane euthanasia was performed if animals developed moribund condition, based on their overall clinical status. Relevant clinical findings may include sustained hypothermia, respiratory distress, >20% weight loss, or neurological signs.

### Humoral and cellular immune assessments

Binding antibody responses against mpox and orthologous vaccinia virus antigens were analysed at baseline and Day 28 using enzyme-linked immunosorbent assays (ELISA) and multiplex electrochemiluminescence assays (ECLA) following established protocols.^21^, ^24^ Cellular immunity was assessed in peripheral blood mononuclear cells (PBMCs) by intracellular cytokine staining after stimulation with vaccinia virus.^21^ Additional information is provided in the Supplementary material.

### Transcriptomics

Blood was collected into PAXgene Blood RNA tubes (BD Biosciences) and RNA was extracted using the MagMAX for Stabilized Blood Tubes RNA Isolation Kit (ThermoFisher Scientific), compatible with PAXgene Blood RNA Tubes. RNA quality was assessed using a 5300 Fragment Analyser (Agilent) and then one microgram of total RNA was subjected to globin transcript depletion using the GLOBINclear Kit, human (ThermoFisher Scientific). Ten nanograms of the globin-depleted RNA were used as input for cDNA synthesis using the SMART-Seq mRNA kit (Takara Bio) according to the manufacturer’s instructions. Amplified cDNA was fragmented and appended with dual-indexed barcodes using the Nextera XT DNA Library Preparation kit (Illumina). Libraries were validated by capillary electrophoresis on a TapeStation 4200 (Agilent), pooled at equimolar concentrations, and sequenced with PE100 reads on an Illumina NovaSeq X Plus, yielding ∼30 million reads per sample on average.

Bulk RNA sequencing was performed on longitudinal blood samples.^21^ Sequencing data was performed at the Emory NPRC Genomics Core and was acquired on an Illumina NovaSeq 6000. Raw counts were normalised using the *edgeR* package and transformed to log2-counts per million (logCPM) with observation-level precision weights estimated using voomWithDreamWeights, which accounts for within-subject correlation by fitting a linear mixed model during the weighting step. All analyses were performed in R (v4.4.2). Differential expression analysis was performed using the *dream* method implemented in the *variancePartition* Bioconductor package, with timepoint as a fixed effect and animal as a random effect to account for repeated measures. Differentially expressed genes (DEGs) were identified using an adjusted *P*-value threshold of 0.05, with *P*-values corrected for multiple testing using the Benjamini-Hochberg procedure. Gene set enrichment analysis was performed using GSEA (v4.4.0) to define molecular signatures from control animals and quantify signature differences across intervention groups. Individual pathway activity was further quantified using single-sample gene set enrichment analysis (ssGSEA), enabling sample-level pathway scoring across the longitudinal cohort. Additional information is provided in the Supplementary material.

### Pharmacokinetic analysis

Trough samples were collected approximately 24 h after tecovirimat dosing, immediately before subsequent dose. Tecovirimat concentrations were quantified using validated HPLC-MS/MS method and were reported as ng/mL, and descriptive statistics were performed using GraphPad Prism (v10.0.0) software.

### Statistics

Lesion counts, plasma viral DNA trajectories, and humoral and cellular responses were summarised in GraphPad Prism (v10.0.0). Peak values and AUC metrics of primary and secondary study endpoints were calculated using the trapezoidal rule. The statistical analysis focused on three comparisons, evaluating each intervention group against the control group using Kruskal–Wallis test with Dunn’s correction for multiple comparisons, and exact *P* values are provided in corresponding figure legends. Non-parametric tests were used because of the small group size and because normality was not assumed. To quantify treatment effect sizes, lesion and viral load AUCs, and T-cell frequencies were log_10_ transformed, and pairwise differences between intervention groups and the untreated control group were reported as Hodges-Lehmann median differences in log_10_ units with non-parametric two-sided 95% confidence intervals. Animals were randomly allocated into groups with balancing for sex and mean group body weight. Clinical lesion assessments were performed by trained personnel using prespecified criteria. No animals were excluded from the analyses after randomisation. For animals euthanised before scheduled study termination, peak values were defined as the maximum observed value before euthanasia, and AUC calculations included all available measurements through the final observation, including unscheduled measurements collected at euthanasia when available; no imputation, last-observation-carried-forward, or extrapolation was performed.

### Role of funders

This study was funded by SIGA Technologies. Authors employed by SIGA Technologies participated in study design, data collection, data analysis and interpretation, and critical review of the report.

## Results

All animals in the intervention groups completed the oral tecovirimat treatment and study follow-up through Day 28. Two of eight animals in the untreated control group met humane euthanasia criteria on Days 16 and 17.

### Early tecovirimat initiation suppresses skin lesions

Consistent with previous studies,^23^, ^25^ control animals infected with mpox showed progressive skin lesions emerge between 5 and 7 days post challenge (Fig. 2; Supplementary Fig. S1; Appendix p 4), with peak lesions between Days 9 and 13 (Supplementary Fig. S2; Appendix p 6). In the tecovirimat-treated groups, skin lesions were highly dependent on the time of treatment initiation. When tecovirimat was started on Day 2 (Group 1), before skin lesions were clinically apparent, lesions were largely prevented, with only 2 of 8 animals developing any progressive skin lesions, and lesion numbers were dramatically reduced (Fig. 2A; Supplementary Figs. S1 and S2; Appendix pp 6–7). We observed markedly lower AUC values for both progressive and total lesion counts (Fig. 2B and C; Supplementary Figs. S3 and S4; Appendix pp 8–9), along with reduced peak lesion counts (Supplementary Fig. S5, Appendix p 10) in Group 1 compared to controls. When tecovirimat was started on Day 5 (Group 2), the early clinical course initially resembled control animals with initial skin lesions appearing on Day 5 in all but one animal, but skin lesions at subsequent timepoints were attenuated compared with controls. Skin lesion counts were significantly reduced between Days 13 and 21 in Group 2 compared with control animals (Supplementary Fig. S2; Appendix p 7). The median AUC values for progressive skin lesions (Fig. 2B and C) were markedly reduced in Group 1 (median 0.0 log_10_; median difference vs. control: −2.81 log_10_, 95% CI −3.20 to −1.94) and Group 2 (median 2.31 log_10_; median difference: −1.54 log_10_, 95% CI −2.30 to −0.62) compared with the control group (median 3.62 log_10_). In contrast, when tecovirimat was started on Day 9 (Group 3), only a modest trend towards reduced AUC values for skin lesions was observed (median 3.05 log_10_; median difference vs. control: −0.79 log_10_, 95% CI −1.84 to 0.01). Total lesion AUC trajectories showed similar patterns (Supplementary Figs. S3 and S4; Appendix pp 8–9), with substantial reductions in Group 1 (median 1.57 log_10_; median difference vs. control: −1.37 log_10_, 95% CI −2.31 to −0.74) and Group 2 (median 2.35 log_10_; median difference: −0.56 log_10_, 95% CI −1.66 to −0.06) compared with controls (median 2.93 log_10_), but no substantial effect in Group 3 (median 2.91 log_10_; median difference vs. control: −0.08 log_10_, 95% CI −1.02 to 0.05). Tecovirimat treatment did not appear to alter the timing of lesion resolution in any group compared to controls, suggesting that time to clinical lesion resolution may be stereotypic and reflective of skin healing and thus not a sensitive endpoint for antiviral efficacy. Two of 8 (25%) animals in the control group met euthanasia criteria for severe disease on Days 16 and 17, while all tecovirimat treated animals survived to the end of the study on Day 28.

**Fig. 2:**
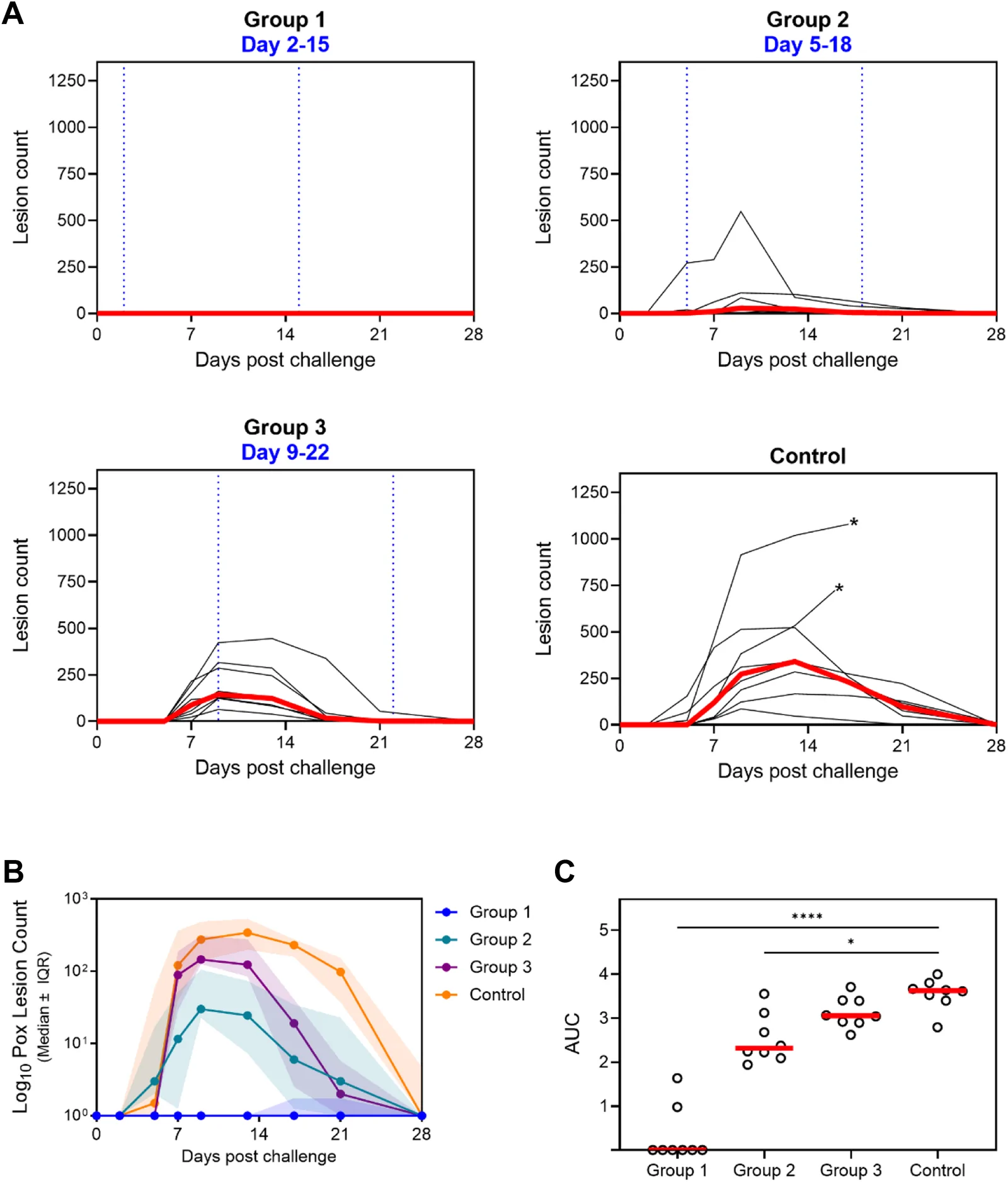
**Progressive skin lesions following mpox challenge.** Skin lesions were quantified on Days 0, 2, 5, 7, 9, 13, 17, 21 and 28 post challenge. Longitudinal lesion counts are shown for each group (**Panel A**), with individual animals represented by solid lines and group medians indicated in red. The tecovirimat treatment period is denoted by blue dotted vertical lines. Two animals in the control group reached humane endpoint criteria and were euthanised on Day 16 and 17 (indicated by asterisks). Group medians of lesion counts (**Panel B**) are shown by coloured lines with shaded areas representing the interquartile range (IQR). Area under the curve (AUC) for lesion counts (**Panel C**) is shown for each animal (circles) on a log-scaled axis, with group medians indicated by red bars. Statistical comparison between intervention groups and the control group were performed using Kruskal–Wallis test with Dunn’s correction for multiple comparisons (∗*P* = 0.0359; ∗∗∗∗*P* < 0.0001).

### Early tecovirimat initiation suppresses plasma viraemia

Plasma viral DNA loads (Fig. 3) were quantified by qRT-PCR and were also highly dependent on the time of treatment initiation. In most animals, plasma viral DNA was detectable on Day 2 after challenge and prior to the appearance of skin lesions. Control animals reached peak viraemia on Day 9. Early tecovirimat initiation on Day 2 (Group 1) was associated with rapid suppression of plasma viraemia to low or undetectable levels in all but one animal by Day 9 (Fig. 3A and B; Supplementary Fig. S6; Appendix p 11). Tecovirimat initiation on Day 5 (Group 2) resulted in a decline of plasma viraemia shortly after treatment initiation with peak viral loads occurring on Day 7 (Fig. 3A and B; Supplementary Fig. S6; Appendix p 11). The median viral load AUC (Fig. 3B and C) were markedly reduced in Group 1 (median 4.53 log_10_; median difference vs. control: −3.10 log_10_, 95% CI −3.99 to −2.12) and Group 2 (median 5.24 log_10_; median difference: −2.13 log_10_, 95% CI −3.01 to −1.07) compared with the control group (median 7.39 log_10_; 95% CI 5.07 to 8.87). Group 2 also exhibited lower viral loads than control animals between Days 9 and 17 (Supplementary Fig. S6; Appendix p 11). Peak viral loads showed similar trends (Supplementary Fig. S7; Appendix p 12). In contrast, tecovirimat initiation on Day 9 (Group 3) did not reduce viral loads (Fig. 3A and B; Supplementary Fig. S6; Appendix p 11), AUC viral loads (median 7.53 log_10_; median difference vs. control: −0.13 log_10_, 95% CI −1.11 to 0.96; Fig. 3B and C), or peak viral loads (Supplementary Fig. S7; Appendix p 12) compared with controls, likely because viral loads had already peaked on Day 9 in the control group.

**Fig. 3:**
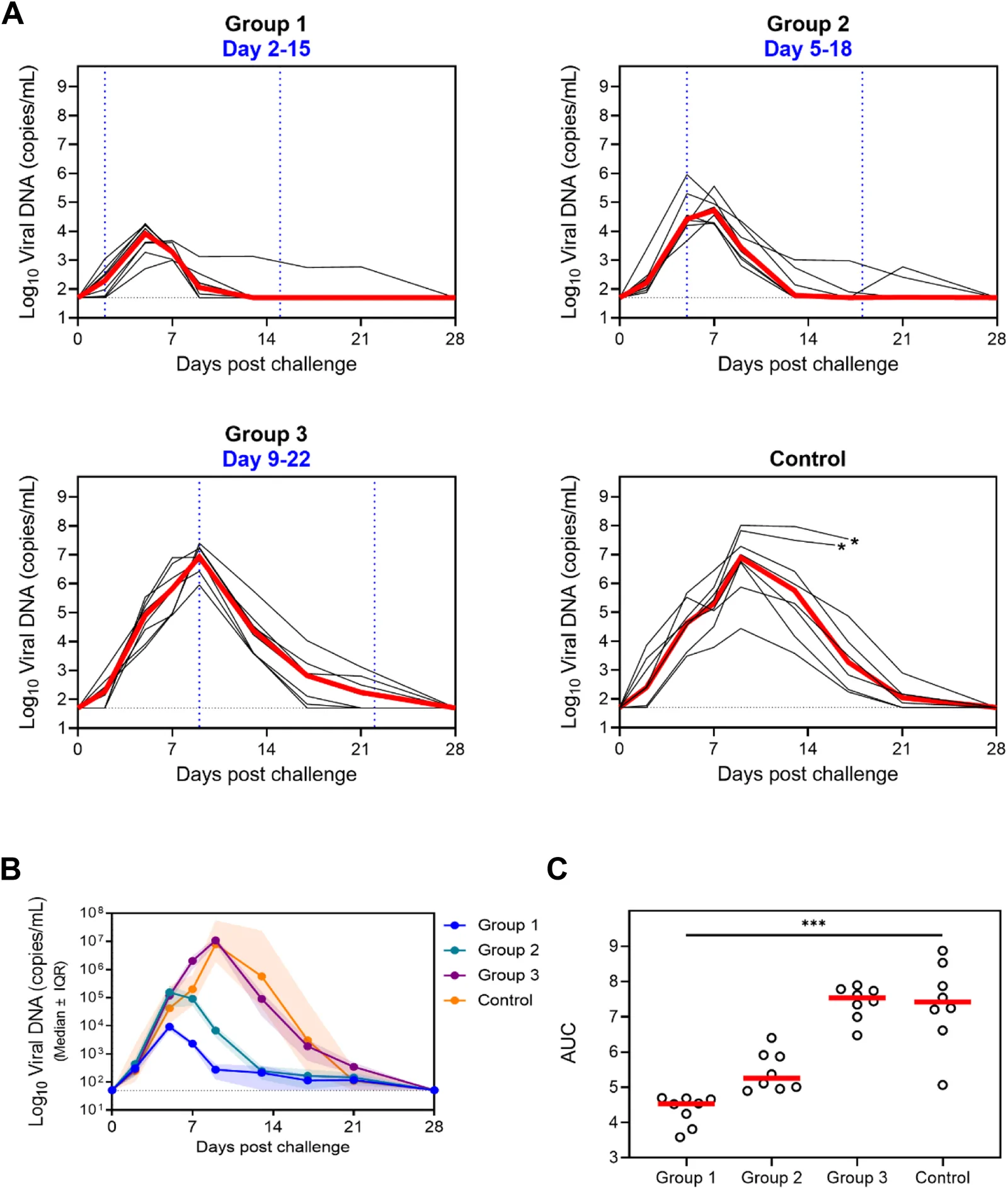
**Plasma viral loads following mpox challenge.** Plasma viral loads (Log10 viral DNA copies/mL) were quantified on Days 0, 2, 5, 7, 9, 13, 17, 21 and 28 post challenge and longitudinal results are shown for each group (**Panel A**). Individual animals are represented by solid lines and group medians are indicated in red. The blue dotted vertical lines indicate the Tecovirimat treatment period, while black dotted lines represent the assay limit of detection (LOD <50 copies/mL). Humane euthanasia in two control animals is indicated by asterisks. The longitudinal group medians (**Panel B**) are shown by coloured lines with IQR indicated by shaded areas. The corresponding area under the viral loads curve (**Panel C**) is shown for each animal (circles) on a log-scaled axis, with group medians reflected by red bars. Statistical differences between intervention groups and the control group were analysed by Kruskal–Wallis test with Dunn’s correction for multiple comparisons (∗∗∗*P* = 0.0001).

These data show that the therapeutic efficacy of tecovirimat following mpox infection was highly dependent on early initiation of treatment after exposure or after appearance of skin lesions. Treatment initiation on Day 9 post challenge, which was 4 days after initial skin lesions and already reflected peak plasma viral loads, was largely ineffective.

### Humoral and cellular immune responses

We next evaluated humoral and cellular immune responses at baseline and on Day 28 following mpox infection. We analysed binding antibody responses to a panel of mpox virus (MPXV) and vaccinia virus (VACV) antigens by ELISA and ECLA assays (Fig. 4A and B). Baseline titres were at or near the assay limit of detection, while antibody responses increased substantially against all antigens tested except M1R/L1R on Day 28. Despite marked suppression of viraemia and lesions in animals in Group 1, antibody responses were still observed in this group but were lower against MPXV B6R by ELISA (Fig. 4A), potentially reflecting decreased antigen exposure to EEV-associated epitopes.^5^

**Fig. 4:**
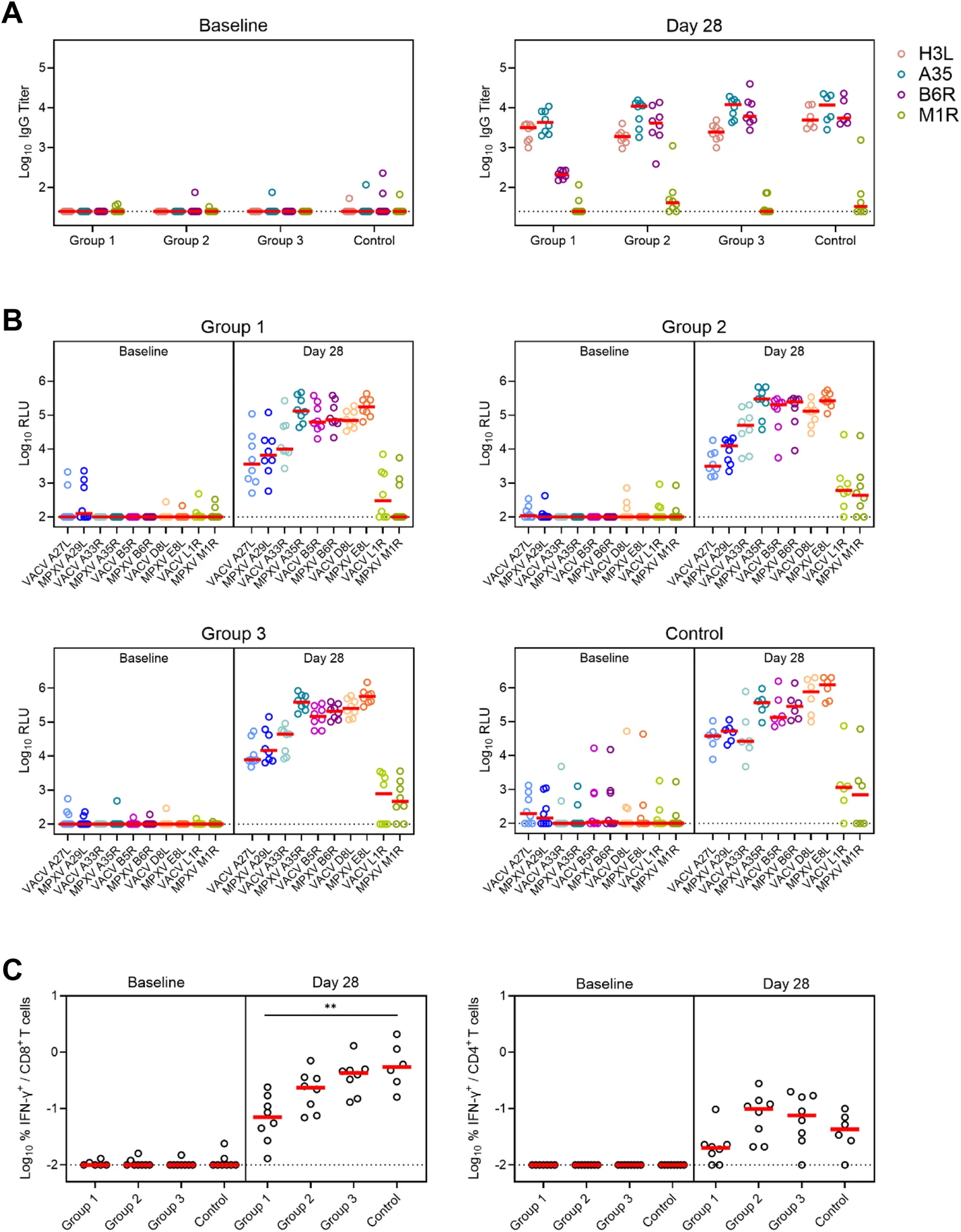
**Humoral and cellular responses.** Humoral and cellular immune responses were assessed prior to challenge (baseline) and at necropsy (Day 28). Due to early humane euthanasia of two animals in the control group, Day 28 samples were only available for six out of eight animals in the control group. Binding antibody responses to selected Mpox antigens were measured by ELISA (**Panel A**). IgG titres for individual animals are shown as circles, with antigens (H3L, A35, B6R, and M1R) displayed in different colours. Red bars represent group median titres for each antigen. The dotted line indicates the limit of detection (LOD < 25). Antibody responses against a broader panel of Mpox and orthologous vaccinia virus antigens were measured using an MSD-ECLA assay (**Panel B**). Individual animals are shown as circles, with red bars indicating group medians. Titres are given in relative light units (RLU) with the dotted line representing the limit of detection (LOD < 100). T cell responses in PBMCs were evaluated following stimulation with vaccinia virus supernatant and IFN-γ intracellular cytokine staining (**Panel C**). Frequencies of IFN-γ^+^ CD8^+^ (left panel) and CD4^+^ (right panel) T cells are shown. Circles represent individual animals and red bars indicate group medians. Dotted lines denote the limit of quantification. Statistical differences between intervention groups and the control group were analysed by Kruskal–Wallis test with Dunn’s correction for multiple comparisons (∗∗*P* = 0.0021).

CD4^+^ and CD8^+^ T cell responses were evaluated by IFN-γ intracellular cytokine staining assays using PBMCs stimulated with vaccinia virus (Fig. 4C). All animals developed virus-specific IFN-γ^+^ CD4^+^ and IFN-γ^+^ CD8^+^ T cell responses. Animals in Group 1 showed significantly lower frequencies of CD8^+^ T cell responses (median −1.15 log_10_; median difference vs. control: −0.90 log_10_; 95% CI −1.47 to −0.34) and a trend towards lower CD4^+^ T cell responses (median −1.69 log_10_; median difference vs. control: −0.31 log_10_; 95% CI −0.77 to 0.15) compared with the control group (CD8^+^ median −0.25 log_10_; 95% CI −0.80 to 0.32; CD4^+^ median −1.38 log_10_; 95% CI −2.00 to −0.92), whereas no statistically significant differences were observed for the other intervention groups compared with the control (Group 2 CD8^+^ median difference: −0.38 log_10_, 95% CI −0.95 to 0.18; CD4^+^ median difference: 0.38 log_10_, 95% CI −0.10 to 0.86; Group 3 CD8^+^ median difference: −0·10 log_10_, 95% CI −0.67 to 0.47; CD4^+^ median difference: 0.24 log_10_, 95% CI −0.24 to 0.72). This lower T cell response in Group 1 likely reflects reduced antigen exposure resulting from early viral suppression.

### Transcriptional pathways following mpox infection

We next performed bulk RNA sequencing to define transcriptomic pathways upregulated and downregulated following mpox infection and impacted by tecovirimat treatment (Fig. 5; Supplementary Figs. S8 and S9; Appendix 13–14). In control animals, mpox infection led to upregulation of multiple inflammatory and cytokine pathways, innate immune pathways, metabolic pathways, and skin repair pathways that peaked on Day 9, concurrent with peak viraemia and consistent with a prior study.^21^ A comparable pattern of responses was observed in animals that started tecovirimat on Day 9 (Group 3). In contrast, initiation of tecovirimat on Day 2 (Group 1) and Day 5 (Group 2) resulted in marked suppression of these transcriptomic signatures of mpox pathogenesis, consistent with the reduced skin lesions and viral loads. Differential gene expression and longitudinal trajectory analyses further supported these findings (Supplementary Figs. S8 and S9).

**Fig. 5:**
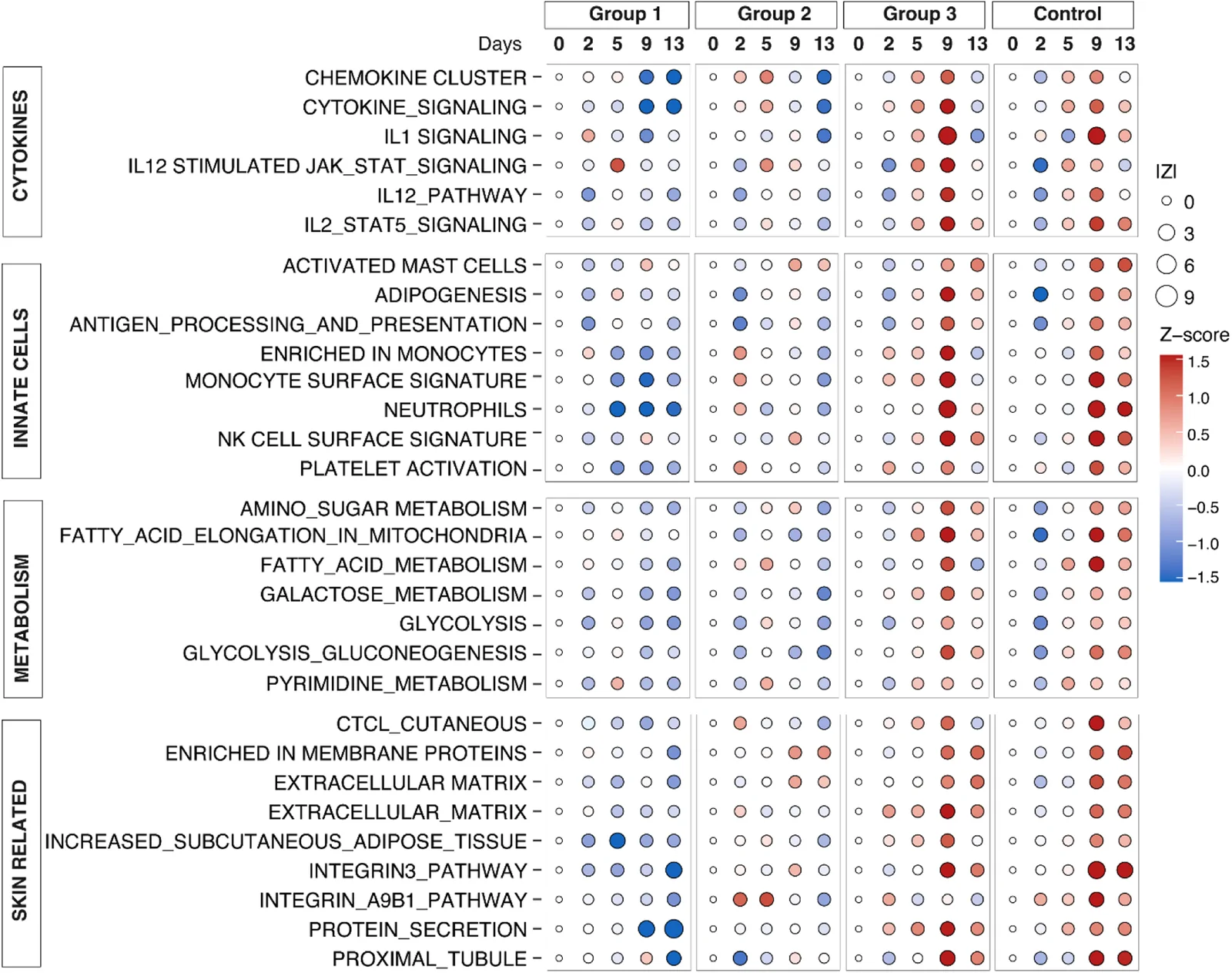
**Transcriptional responses following mpox challenge.** Bubble plot showing single-sample gene set enrichment analysis (ssGSEA) normalised enrichment scores (NES) across four experimental groups (Group 1, Group 2, Group 3, and Control) and five longitudinal timepoints (Days 0, 2, 5, 9, and 13 post–challenge). Gene sets were organised into four biological modules: Proinflammatory Response, Skin-Related, Innate Cells, and Metabolism. Bubble colour reflects the z-score of the NES (red = upregulation, blue = downregulation; scale −1.5 to +1.5), and bubble size reflects the absolute z-score (|Z|, ranging from 0 to 9). Each column represents a timepoint within a group; Day 0 corresponds to the pre-infection baseline (wk−6). Pathway activity was assessed relative to baseline using normalised enrichment scores computed at the individual sample level and then summarised per group and timepoint.

### Tecovirimat drug levels

Systemic tecovirimat exposure during the treatment period was confirmed by measuring trough plasma concentrations approximately 24 h after dosing. Geometric mean concentrations were comparable across all intervention groups with 176.1 ng/mL (95% CI 149.2 to 207.8) in Group 1, 190.8 ng/mL (95% CI 149.2 to 244.1) in Group 2, and 151.0 ng/mL (95% CI 114.7 to 198.8) in Group 3, and an overall geometric mean of 171.9 ng/mL (95% CI 152.8 to 193.4) across all intervention groups (Supplementary Table S2).

## Discussion

Tecovirimat therapeutic efficacy against clade IIb mpox infection in macaques was highly dependent on the timing of treatment initiation. Post-exposure and early initiation of treatment on Day 2 or Day 5 prior to peak viraemia resulted in significant reductions in skin lesions, viral loads, and transcriptomic signatures of mpox pathogenesis, whereas initiation of treatment on Day 9 post challenge, which was 4 days after initial appearance of skin lesions and already reflected peak plasma viral loads, was largely ineffective. These data demonstrate the critical importance of early initiation of tecovirimat therapy following mpox infection prior to peak viraemia in nonhuman primates.

The requirement for early initiation of tecovirimat is consistent with other antiviral therapeutics, such as oseltamivir for influenza and nirmatrelvir/ritonavir for COVID-19, which similarly require initiation of treatment within 2–3 days of symptoms. For mpox in macaques, our data suggest a therapeutic window of less than 4 days following appearance of skin lesions. We suspect a similar therapeutic window may exist in humans, although clinical efficacy trials will be required to confirm these results. These results are also consistent with prior preclinical tecovirimat studies,^9^, ^11^, ^12^, ^13^, ^14^ which have demonstrated robust therapeutic efficacy when tecovirimat was given early after orthopoxvirus challenge.^9^, ^14^ The present study extends these prior observations in a nonlethal mpox clade IIb challenge model that more closely reflects the typical self-limited clinical course of mpox clade IIb in most humans.^19^, ^20^, ^26^

Our data also provide a framework to interpret the lack of tecovirimat therapeutic efficacy in the recent STOMP and PALM007 clinical trials. In STOMP, participants were enrolled at a median of 8 days after symptom onset.^10^ In PALM007, participants were enrolled at a median of 6 days after symptom onset with an average of 487 skin lesions.^18^ Tecovirimat did not shorten time to lesion resolution in either study. Our data suggest that these studies likely enrolled patients beyond the therapeutic window for tecovirimat efficacy. In our macaque study, tecovirimat was only minimally effective on Day 9 post challenge, which was 4 days after onset of skin lesions. We therefore speculate that tecovirimat was started too late in STOMP and PALM007, likely after viraemia had peaked and when viral replication was already being controlled by host immune responses and when skin lesions were already in the healing process. Moreover, the time to resolution of skin lesions was not reduced in any treatment group in our study, suggesting that skin lesion resolution is largely stereotypic and reflective of skin healing processes rather than active viral replication.

Our data suggest that tecovirimat has therapeutic efficacy for mpox but only when it is started before or shortly after appearance of skin lesions.^27^ Future clinical trials should therefore consider evaluating earlier initiation of tecovirimat after onset of symptoms, although early treatment may be a challenge in clinical practice, which highlights the importance of rapid and sensitive point-of-care diagnostics. Our findings also suggest that tecovirimat should be evaluated for post-exposure prophylaxis. This strategy may be particularly valuable for high-risk contacts, people who are immunocompromised, or after presumed exposure in outbreak scenarios, enabling immediate treatment prior to symptom onset, although validating its efficacy will necessitate future dedicated clinical trials. More broadly, these findings highlight the importance of aligning animal efficacy studies and subsequent human clinical trials, when feasible, with respect to the biological stage of disease at treatment initiation and study endpoints.

The main limitation of this study is that it uses macaques rather than humans to evaluate the impact of timing of tecovirimat initiation on therapeutic efficacy. High-dose intravenous challenge in nonhuman primates produces a rapid, synchronised systemic infection with peak viraemia and lesions occurring within 6–9 days, whereas natural human exposure typically occurs through mucosal or cutaneous surfaces, often leading to a longer incubation period and viral dynamics concentrated in skin or mucosal tissues. Consequently, these time effects define biological stage-dependent efficacy and thus Day 2 represents post-exposure, pre-symptomatic intervention rather than a direct calendar day equivalent in human clinical presentation. Moreover, we used numbers of skin lesions and plasma viral loads as endpoints rather than time to resolution of skin lesions. Strengths of this study include the randomised, controlled study design with prespecified treatment initiation at three distinct biological stages of Mpox infection. The longitudinal assessment of clinical, virological, immunological, transcriptional changes and pharmacokinetics allowed treatment effect to be evaluated across multiple measures of disease.

In conclusion, this study shows that tecovirimat needs to be initiated rapidly after mpox infection and prior to peak viraemia and peak skin lesions for optimal therapeutic efficacy in macaques. This study also provides an explanation for the discordance between the positive results of the prior macaque studies that led to regulatory approval by the Animal Rule and the recent negative results of the STOMP and PALM007 clinical trials.

## Contributors

Drs Rössler, Russo, Grosenbach, Hruby, and Barouch had full access to all data in the study and take responsibility for the integrity of the data and the accuracy of the data analysis. All authors read and approved the final version of the manuscript.

*Concept and design:* Rössler, Russo, Grosenbach, Hruby, Barouch.

*Acquisition, analysis and interpretation of the data:* All authors.

*Drafting of the manuscript:* Rössler, Barouch.

*Critical review of the manuscript for important intellectual content:* All authors.

*Statistical analysis:* Rössler, Aid.

*Administrative, technical, or material support:* All authors.

*Supervision:* Hruby, Andersen, Barouch.

## Data sharing statement

The RNA-sequencing datasets generated in this study have been deposited in the NCBI Gene Expression Omnibus (GEO) under accession number GSE341361. Other data are available upon request from the corresponding author (dbarouch@bidmc.harvard.edu).

## Declaration of interests

A.T.R., C.L. and D.E.H. hold stock in SIGA Technologies, Inc., and D.E.H additionally holds patents related to tecovirimat. A.R., M.A., J.A.B., J.L., A.C., H.A, D.W.G., and D.H.B. declare no competing interests.
