# Supplementary material for: Early treatment with tecovirimat against mpox disease in cynomolgus macaques
Source: eBioMedicine. 2026 Oct 21;133:106526. doi: 10.1016/j.ebiom.2026.106526 (PMC13715304; doi:10.1016/j.ebiom.2026.106526)
Supplement: Supplementary file 1 [file mmc1.docx]

**Supplementary Material**

**Plasma viral-load quantification**

Mpox viral DNA in plasma samples was quantified by qRT-PCR targeting the F3L gene (GenBank accession URK20488) as previously described^1,2^. Viral DNA was extracted using the QIAamp 96 DNA Blood Kit on a QIAcube HT (Qiagen), following the manufacturer’s instructions. For the amplification F3L-specific primers (F3L.FWD: CATCTATTATAGCATCAGCATCAGA; F3L.REV: GATACTCCTCCTCGTTGGTCTAC; Integrated DNA Technologies) and a BHQ-labeled probe (FAM-TGTAGGCCGTGTATCAGCATCCATT-BHQ; Biosearch Technologies) were used on QuantStudio 6 and 7 Flex systems (Applied Biosystems). Cycling conditions were 95°C for 20 seconds followed by 45 cycles of 95°C for 1 second and 60°C for 20 seconds. Absolute quantification used an in-house DNA standard spanning 10^10^ to 10^-1^ copies to generate a standard curve and report viral DNA copies per mL, with a limit of detection of 50 copies/mL.

**Serologic assays**

Binding antibody titres to Mpox antigens were measured by enzyme-linked immunosorbent assays (ELISA) as previously described ^1,2^. Plates were coated overnight with 1 µg/mL antigen (M1R, A35, H3L, or B6R; Sino Biologicals). Heat-inactivated sera were serially diluted (3-fold starting at 1:25), incubated for 1 hour at room temperature, washed, and detected using an anti-macaque IgG–HRP conjugate (NIH Nonhuman Primate Reagent Resource). Assays were developed with KPL TMB SureBlue substrate (SeraCare) and read at 650 nm and 450 nm on a VersaMax plate reader (Molecular Devices). Endpoint titres were derived by 4-parameter logistic curve fitting in GraphPad Prism (v10.0.0).

Serum IgG reactivity against a broader orthopox antigen panel was measured using the multiplex electrochemiluminescence assay (ECLA) V-PLEX Orthopoxvirus Panel 1 (IgG) kit (Meso Scale Discovery; Cat. K15688U) following the manufacturer’s protocol and as described previously ^1,2^. The panel included the MPXV/VACV orthologous antigens M1R/L1R, A28/A30L, A27L/A29L, A33R/A35R, B5/B6R, and D8L/E8L. Plates were read on a MESO QuickPlex SQ 120, and results were reported as relative light units (RLU).

**Intracellular cytokine staining**

Peripheral blood mononuclear cells (PBMCs) collected at baseline and necropsy (1x10^6^ cells) were stimulated as previously described^2^. Cells were resuspended in medium containing CD49d (1 µg/mL) and stimulated with mock control (R10 + 0.5% DMSO), vaccinia virus Western Reserve (1x10^7^ viral particles; ATCC VR-1354), or PMA (10 pg/mL) plus ionomycin (1 µg/mL). After 3 hours at 37°C, GolgiStop and GolgiPlug were added, followed by 6 additional hours at 37 °C and overnight storage at 4 °C. Cells were stained with a near-IR viability dye, followed by surface staining (including CD279, CD38, CD28, CD4, CD45, CD95, CD8), fixed/permeabilised, and stained intracellularly (including Ki67, CD69, IL-10, IL-13, TNF-α, IL-4, IFN-γ, IL-2, CD3). Samples were fixed in 1.5% formaldehyde and acquired on a BD FACSymphony flow cytometer.

**Bulk RNA-seq and transcriptomic analyses.**

Reads were aligned and quantified using STAR (v2.7.9a) against a combined reference consisting of the Mmul10 macaque genome (Ensembl v100 annotations) and the ACM1462154v1 Mpox genome/annotation^3^. Longitudinal differential expression analysis were performed separately within each group using linear mixed models in the DREAM framework^4^.

Counts were transformed with voomWithDreamWeights to model mean–variance structure and generate observation-level precision weights. Timepoint was modeled as a fixed effect (pre-infection baseline, week 6 prior challenge (referred to as wk-6), as the reference), and animal identity was included as a random intercept (~ Timepoint + (1 | NHP)). Empirical Bayes moderation was applied, and p-values were adjusted using the Benjamini–Hochberg procedure. Differential expression at each timepoint was defined relative to baseline within group.

To test whether early treatment in Group 1 (Day 2) prevented establishment of an infection-associated transcriptional program, an infection signature was defined in control animals by comparing gene expression at peak viraemia to pre-infection baseline. Genes meeting FDR < 0.05 were used as the predefined signature. Signature expression was then evaluated in the Day 2 treatment group at the corresponding chronological timepoint using pathway enrichment and gene set scoring approaches. Groups treated on Days 5 (Group 2) and 9 (Group 3) were evaluated in secondary analyses to assess how delayed initiation altered modulation of infection-associated pathways.

GSEA was performed on preranked gene lists derived from differential expression statistics. Enrichment scores were computed using the standard GSEA running-sum approach, and significance was assessed with permutation testing and FDR correction. In parallel, ssGSEA was used to calculate normalised enrichment scores for predefined signatures at the individual-sample level to enable longitudinal tracking of pathway activity.

**Supplementary Table 1: Reagents**

| **Reagent or Resource** | **SOURCE** | **IDENTIFIER** |
| --- | --- | --- |
| **Antibodies and Staining Reagents** | | |
| Anti-human/NHP CD3 (clone SP34.2, Alexa Fluor 700) | BD Biosciences | Cat# 557917; RRID: AB_396937 |
| Anti-human/NHP CD4 (clone L200, BV510) | BD Biosciences | Cat# 563914; RRID: AB_2738485 |
| Anti-human/NHP CD8 (clone SK1, BUV805) | BD Biosciences | Cat# 612889; RRID: AB_2870176 |
| Anti-human/NHP CD28 (clone 28.2, PE-Cy5) | BD Biosciences | Cat# 555730; RRID: AB_396071 |
| Anti-human/NHP CD45 (clone D058-1283, BUV615) | BD Biosciences | Cat# 613010; RRID: AB_2870233 |
| Anti-human/NHP CD69 (clone TP1.55.3, ECD) | Beckman Coulter | Cat# 6607110; RRID: AB_1575968 |
| Anti-human/NHP CD95 (clone DX2, BUV737) | BD Biosciences | Cat# 612790; RRID: AB_2870118 |
| Anti-human/NHP CD279 / PD-1 (clone EH12.1, BB700) | BD Biosciences | Cat# 565281; RRID: AB_2739151 |
| Anti-human/NHP Ki67 (clone B56, FITC) | BD Biosciences | Cat# 556026; RRID: AB_396302 |
| Anti-human/NHP IFN-g (clone B27, BUV395) | BD Biosciences | Cat# 563563; RRID: AB_2738276 |
| Anti-human/NHP IL-2 (clone MQ1-17H12, APC) | BD Biosciences | Cat# 561054; RRID: AB_10562551 |
| Anti-human/NHP IL-4 (clone MP4-25D2, BV711) | BD Biosciences | Cat# 564114; RRID: AB_2738604 |
| Anti-human/NHP IL-10 (clone JES3-9D7, PE-Cy7) | BioLegend | Cat# 501410; RRID: AB_315162 |
| Anti-human/NHP IL-13 (clone JES10-5A2, BV421) | BD Biosciences | Cat# 563580; RRID: AB_2738289 |
| Anti-human/NHP TNFa (clone Mab11, BV650) | BD Biosciences | Cat# 563418; RRID: AB_2738190 |
| Anti-human/NHP CD49d monoclonal antibody (pure) | BD Biosciences | Cat# 555501; RRID: AB_395888 |
| Anti-human/NHP CD28 monoclonal antibody (pure) | BD Biosciences | Cat# 555725; RRID: AB_396068 |
| **Viruses** | | |
| Monkeypox virus (hMPXV/USA/MA001/2022, lineage B.1, clade IIb) | BEI Resources | Cat# NR-58622 |
| Vaccinia virus (Western Reserve strain) | ATCC | Cat# VR-1354 |
| **Cell Lines** | | |
| MA-104 (Clone 1; African green monkey kidney cells) | ATCC | Cat# CRL-2378.1 |
| Vero E6 (African green monkey kidney cells) | ATCC | Cat# CRL-1586; RRID: CVCL_0574 |
| **Chemicals and biological Reagents** | | |
| Tecovirimat (ST-246) | SIGA Technologies | N/A |
| Phorbol 12-myristate 13-acetate (PMA) | Sigma-Aldrich | Cat# P8139 |
| Ionomycin calcium salt | Sigma-Aldrich | Cat# I0634 |
| Dimethyl sulfoxide (DMSO) | Sigma-Aldrich | Cat# D8418 |
| Formaldehyde Solution (1.5% diluted in DPBS) | Sigma-Aldrich | Cat# F8775 |
| Phosphate-Buffered Saline (PBS), pH 7.4 | ThermoFisher Scientific | Cat# 10010023 |
| **Commercial Assays and Kits** | | |
| PAXgene Blood RNA Tubes | BD Biosciences | Cat# 762165 |
| MagMAX for Stabilized Blood Tubes RNA Isolation Kit | ThermoFisher Scientific | Cat# 4451894 |
| GLOBINclear Kit, human | ThermoFisher Scientific | Cat# AM1980 |
| SMART-Seq mRNA Kit | Takara Bio | Cat# 634771 |
| Nextera XT DNA Library Preparation Kit | Illumina | Cat# FC-131-1024 |
| LIVE/DEAD Fixable Near-IR Dead Cell Stain Kit | Life Technologies / ThermoFisher | Cat# L10119 |
| BD CytoFix/CytoPerm Fixation/Permeabilization Kit | BD Biosciences | Cat# 554714 |
| BD GolgiStop Protein Transport Inhibitor (Monensin) | BD Biosciences | Cat# 554724 |
| BD GolgiPlug Protein Transport Inhibitor (Brefeldin A) | BD Biosciences | Cat# 555029 |
| BD Perm/Wash Buffer (10x) | BD Biosciences | Cat# 554723 |
| **Deposited Data and Instrumentation** | | |
| Bulk RNA-sequencing Dataset | NCBI Gene Expression Omnibus (GEO) | Accession: GSE341361 |
| BD FACSymphony Cell Analyzer | BD Biosciences | N/A |
| 5300 Fragment Analyzer | Agilent Technologies | N/A |
| Illumina NovaSeq X Plus / NovaSeq 6000 | Illumina | N/A |
| **Software and Algorithms** |  |  |
| R Programming Environment (v4.4.2) | R Foundation for Statistical Computing | [https://www.R-project.org/](https://www.r-project.org/); RRID: SCR_001905 |
| edgeR Bioconductor package | Bioconductor | <https://bioconductor.org>; RRID: SCR_012802 |
| variancePartition Bioconductor package (dream) | Bioconductor | <https://bioconductor.org>; RRID: SCR_019230 |
| Gene Set Enrichment Analysis (GSEA, v4.4.0) | Broad Institute | <https://www.gsea-msigdb.org>; RRID: SCR_003199 |
| FlowJo Software (v10.8.1) | FlowJo, LLC / BD | <https://www.flowjo.com>; RRID: SCR_008520 |
| GraphPad Prism (v10.0.0) | GraphPad Software | <https://www.graphpad.com>; RRID: SCR_002798 |

**Supplementary Table 2: Summary of tecovirimat trough serum concentrations across intervention groups**

| **Statistic** | **Group 1** | **Group 2** | **Group 3** | **Overall^a^** |
| --- | --- | --- | --- | --- |
| N (samples) | 16 | 16 | 16 | 48 |
| Geometric mean | 176.1 | 190.8 | 151.0 | 171.9 |
| Geometric %CV | 31.8 | 48.8 | 55.3 | 46.2 |
| Mean ± SD (ng/mL) | 184.1 ± 56.1 | 211.3 ± 102.6 | 170.8 ± 95.4 | 188.8 ± 87.0 |
| Median (ng/mL) | 166.5 | 191.0 | 161.5 | 165.5 |
| Minimum (ng/mL) | 103 | 99.2 | 43.1 | 43.1 |
| Maximum (ng/mL) | 284 | 447 | 471 | 471 |
| ^a^Overall values calculated from 48 individual trough values, not from group means. | | | | |

**Supplementary Figure 1. First appearance of skin lesions following mpox challenge**

Skin lesions were quantified at defined intervals throughout the study (Day 2, 5, 7, 9, 13, 17, 22 and 28 post challenge). The day of first detection of progressive skin lesions for each animal is shown as circles, with group medians indicated by red bars. The total number of animals per group (n=8) that developed skin lesions is indicated above each group. Only 2 of 8 animals in Group 1 developed skin lesions after mpox challenge. Statistical comparison between intervention groups and control group was performed by Kruskal–Wallis test with Dunn’s correction for multiple comparisons (**P*=0.0479).

**Supplementary Figure 2. Comparative progressive skin lesions following mpox challenge**

Skin lesion counts were assessed on indicated days following mpox challenge. Progressive skin lesion counts at each timepoint are shown, with circles representing individual animals and red bars indicating group medians. Due to early humane euthanasia of two animals in the control group on Day 16 and 17, later timepoints were not available for all animals of the control group. Statistical differences between intervention groups and control group were analysed by Kruskal–Wallis test with Dunn’s correction for multiple comparisons. Only significant comparisons are shown: Day 7, ****P*=0.0004; Day 9, *****P*<0.001; Day 13, *****P*<0.0001*,* **P*=0.0124; Day 17, *****P*<0.0001, **P*=0.0203; Day 21, ***P*=0.0012*,* Group 2 vs. Control, **P*=0.0456, Group 3 vs. Control, **P*=0.0321*)*

**Supplementary Figure 3. Total skin lesions following mpox challenge**

Total skin lesions were quantified on Days 0, 2, 5, 7, 9, 13, 17, 22 and 28 post challenge. Longitudinal lesion counts are shown for each group (**Panel A**), with individual animals represented by solid lines and group medians indicated in red. The tecovirimat treatment period is denoted by blue dotted vertical lines. Two animals in the control group reached humane endpoint criteria and were euthanised on Day 16 and 17 (indicated by asterisks). Group medians of lesion counts (**Panel B**) are shown by coloured lines with shaded areas representing the interquartile range (IQR). Area under the curve (AUC) for lesion counts (**Panel C**) is shown for each animal (circles) on a log-scaled axis, with group medians indicated by red bars. Statistical comparison between intervention groups and control were performed using Kruskal–Wallis test with Dunn’s correction for multiple comparisons (*****P*<0.0001, **P*=0.0424).

**Supplementary Figure 4. Comparative total skin lesions following mpox challenge**

Total skin lesion counts were assessed on indicated days following mpox challenge. Total skin lesion counts at each timepoint are shown, with circles representing individual animals and red bars indicating group medians. Due to early humane euthanasia of two animals in the control group on Day 16 and 17, later timepoints were not available for all animals of the control group. Statistical differences between intervention groups and control were analysed by Kruskal–Wallis test with Dunn’s correction for multiple comparisons. Only significant comparisons are shown: Day 5, ***P*=0.0010; Day 7, ****P*=0.0003; Day 9, *****P*<0.001; Day 13, *****P*<0.0001*,* **P*=0.0199; Day 17, *****P*<0.0001 **P*=0.0239; Day 21, ****P*=0.0008).

**Supplementary Figure 5. Peak progressive and total skin lesion counts**

Skin lesions were quantified at defined timepoints, and maximum (peak) counts of progressive lesions (**Panel A**) and total lesions (**Panel B**) for each animal are shown. Circles represent individual animals, and red bars indicate the group median. The number of animals per group (n=8) that developed skin lesions is indicated by numbers above each group. Statistical differences between intervention groups and control were analysed by Kruskal–Wallis test with Dunn’s correction for multiple comparisons (*****P*<0.0001).

**Supplementary Figure 6. Comparative plasma viral loads following mpox challenge**

Plasma viral loads (Log_10_ viral DNA copies/mL) were assessed on indicated days following mpox challenge. Viral loads at each timepoint are shown, with circles representing individual animals and red bars indicating group medians. Due to early humane euthanasia of two animals in the control group on Day 16 and 17, later timepoints were not available for all animals of the control group. Statistical differences between intervention groups and control analysed by Kruskal–Wallis test with Dunn’s correction for multiple comparisons. Only significant comparisons are shown: Day 5, **P*=0.0310*;* Day 7, ***P*=0.0012; Day 9, *****P*<0.0001*,* **P*=0.0249; Day 13, *****P*<0.0001*,* ***P*=0.0011; Day 17, Group 1 vs. control, ***P*=0.0011, Group 2 vs. control, ***P*=0.0045).

**Supplementary Figure 7. Peak plasma viral loads following mpox challenge**

Plasma viral loads (Log_10_ viral DNA copies/mL) were quantified at defined timepoints, and maximum (peak) viral loads for each animal are shown. Circles represent individual animals, and red bars indicate the group median. The dotted line denotes the limit of detection (LOD <50 copies/mL). Statistical comparison between intervention groups and control were performed using Kruskal–Wallis test with Dunn’s correction for multiple comparisons (****P*=0.0001).

**Supplementary Figure 8. Differentially expressed genes across timepoints.**

UpSet plots displaying the intersections of differentially expressed genes (DEGs) across timepoints post challenge (Day 2, 5, 9, and 13) for each of the four experimental groups: Group 1, Group 2, Group 3, and the control group. Horizontal bars (left) represent the total number of DEGs detected at each timepoint (set size). Vertical bars indicate the size of each intersection, with connected dots below identifying which timepoints contribute to that intersection. DEGs were identified by longitudinal differential expression analysis using linear mixed models implemented in the DREAM framework, with the pre-infection baseline (wk−6) as reference and animal identity included as a random intercept to account for repeated measures. Statistical significance was defined at FDR < 0.05 after Benjamini–Hochberg correction.

**Supplementary Figure 9. Gene-level transcriptional trajectories across timepoints.**

Z-score heatmaps displaying longitudinal expression of the infection transcriptional signature for each experimental group (Group 1, Group 2, Group 3, control) across five timepoints (Days 0, 2, 5, 9, and 13 post-infection). Each row represents an individual gene and each column a given timepoint, ordered chronologically. The infection signature was defined as genes differentially expressed (FDR <0.05) in untreated animals at peak viraemia relative to their pre-infection baseline. Expression values were z-score normalised per gene within each group. Colour indicates the direction and magnitude of expression change (red = upregulation, blue = downregulation; white = no change). Genes are ordered alphabetically and a fixed colour scale is applied across all groups to enable direct cross-group comparison.

**Supplementary References**

1.	Aid M, Sciacca M, McMahan K, et al. Mpox infection protects against re-challenge in rhesus macaques. Cell 2023;186(21):4652–4661.e13. (In eng). DOI: 10.1016/j.cell.2023.08.023.

2.	Jacob-Dolan C, Ty D, Hope D, et al. Comparison of the immunogenicity and protective efficacy of ACAM2000, MVA, and vectored subunit vaccines for Mpox in rhesus macaques. Sci Transl Med 2024;16(740):eadl4317. (In eng). DOI: 10.1126/scitranslmed.adl4317.

3.	Medicine N-NLo. Genome assembly ASM1462154v1. (<https://www.ncbi.nlm.nih.gov/datasets/genome/GCF_014621545.1/>).

4.	Hoffman GE, Roussos P. Dream: powerful differential expression analysis for repeated measures designs. Bioinformatics 2021;37(2):192–201. (In eng). DOI: 10.1093/bioinformatics/btaa687.
